# Research on the Status of Intangible Cultural Heritage Bearers in the Human Capital Perspective

**DOI:** 10.3389/fpsyg.2022.850780

**Published:** 2022-03-30

**Authors:** Jing Zhao, Zhong Wang, Chenyu Wang, Liming Han, Yaohui Ruan, Zhounan Huangfu, Shuai Zhou, Lei Zhou

**Affiliations:** ^1^Design College, Zhoukou Normal University, Zhoukou, China; ^2^Faculty of Humanities and Social Sciences, City University of Macau, Macau, Macau SAR, China; ^3^Faculty of Innovation and Design, City University of Macau, Macau, Macau SAR, China; ^4^Fashion college, Changzhou Vocational Institute of Textile and Garment of Changzhou, Changzhou, China; ^5^Faculty of Fine Arts, Srinakharinwirot University, Bangkok, Thailand; ^6^Valaya Alongkorn Rajabhat University, Bangkok, Thailand; ^7^Asia-Europe Institute, University of Malaya, Kuala Lumpur, Malaysia; ^8^Art Design College, Henan University of Engineering, Zhengzhou, China

**Keywords:** human capital, social capital, psychological capital, career identity, job satisfaction, entrepreneurship ability, status attainment

## Abstract

Culture is the bloodline of the nation and the spiritual home of the people. Intangible cultural heritage (ICH) belongs to the field of culture, and the transmission of ICH is a kind of human-based cultural transmission, which is the shaping of people’s morality, character, sentiment, will, ideals and beliefs, value orientation, humanistic cultivation, artistic taste, way of thinking, wisdom, and ability in the practice of production and life of various ethnic groups. Based on the status acquisition model, this study analyzed the human capital (HC), social capital (SC), and psychological capital (PC) of ICH bearers from three perspectives. In addition to the conventional socio-demographic factors such as gender, age, place of residence, and education level as control variables, status attainment was introduced as a dependent variable, and occupational identity, job satisfaction, and entrepreneurial ability were introduced as mediating variables to construct a conceptual model in the hope of exploring the multiple influencing factors of status attainment among ICH bearers. Interviews were used to gain, the feelings and knowledge of experts, scholars, and ICH bearers, to lay a solid qualitative research foundation for this study. A questionnaire survey was also conducted to obtain basic information and professional experiences of ICH bearers to provide real support for the research analysis and discussion. As per the results of this research, all the hypotheses were supported except, HC did not have any significant impact on JS. Furthermore, SC was not in a significant association with career identity. Moreover, career identity and status attainment did not have a significant relationship.

## Introduction

Culture is the bloodline of the nation and the spiritual home of the people. Intangible cultural heritage (ICH) belongs to the field of culture, and the transmission of ICH is a kind of human-based cultural transmission, which is the shaping of people’s morality, character, sentiment, will, ideals and beliefs, value orientation, humanistic cultivation, artistic taste, way of thinking, wisdom, and ability in the practice of production and life of various ethnic groups (Wei et al., 2021). It is the most basic, deepest, and most lasting cultural spirit that a nation depends on for survival. This core value is a collection of social value orientation and personal value orientation, directly reflecting the strength of a country’s cultural soft power, the formation process of this value constitutes the fundamental ideological impetus for the enhancement of the country’s cultural soft power (Lenzerini, 2011). In-depth knowledge and understanding of heritage, and in-depth research on heritage, is the key to good ICH protection work. As the core carrier of ICH protection, ICH bearers are still the key area of ICH research in the new era (Petronela, 2016). This study attempts to apply the basic framework of three-dimensional capital theory comprising human, psychological, and social capital to the field of sociological research to analyze the transmission and protection of ICH.

This research examines the association of human, psychological and social capital with the career identity, job satisfaction, and entrepreneurship ability of ICH bearers. The human capital theory offers a theoretic foundation for understanding the specific methodology to career accomplishment. Human capital theory (1976) implies that people that spend the highest in human capital (HC) aspects for instance learning, education, and knowledge are likely to exhibit a greater degree of job execution and consequently achieve greater organizational incentives. Corresponding to this concept, a person’s career development and achievement are reliant upon the number and value of human resources an individual gives to the job market (Agarwal, 1981; Ballout, 2007). Hence, this study indicates an association between human capital and career identity. Career identity is described as the approach by which individuals identify themselves in the perspective of their career, working as an intellectual scope utilized to route career prospects (Fugate et al., 2004). Likewise, earlier findings similarly suggest that job performance and satisfaction are impacted by HC (Allen and Van der Velden, 2001; Langelett, 2002; Lyons and Akroyd, 2014). Therefore, this study analyzes the association of HC with job satisfaction. Additionally, the concern in HC in the entrepreneurship narrative is established and has risen around the past twenty years (Marvel et al., 2016). The concept has been progressively employed in the sphere of entrepreneurship, constantly connecting HC characteristics to entrepreneurial achievement (Unger et al., 2011). Consequently, this study examines the relationship of human capital with entrepreneurship ability.

Luthans and Youssef (2004) suggested the idea of psychological capital (PC) in the context of constructive psychology and organizational conduct. They described PC as a certain attitude demonstrated throughout the development and growth of a person. It comprises four fundamental elements of optimism, self-efficacy, hope, and resilience (Peng et al., 2013). The four fundamental concepts of PC are significantly linked to mindsets and feelings of workers which can influence their conduct or behavior intents (2010). The PCs are usually discovered to possess enormous influence toward delivering competitive benefits of a company because it is challenging to replicate by the competition (Tang, 2020). The PC is commonly observed to affect the conduct and attitude of workers that additionally, has an immediate effect on enhancing organizational functioning (Newman et al., 2014). According to positive psychology, the higher the PC of ICH inheritors, the higher their positive occupational identity. They are also more likely to dedicate additional energy and time to their job. They also devote more time and energy to develop their potential, stimulate the joy of ICH transmission, and make positive attributions (Luthans et al., 2005). Hence, this research paper attempts to analyze the relationship of PC with career identity. Furthermore, this research explores the impact of PC on job satisfaction (Sun et al., 2012), and, entrepreneurship ability (Tang, 2020).

The rising attention in social capital (SC), remarkably in the progressively uneven and particular academic world, a comprehensive range of academic disciplines, comprising sociology, psychology (human resource) management, industrial relations, and history, economics, anthropology, political theory, and organization studies (Lange, 2015). SC has been defined as the number of philanthropic inclinations and the degree of communal trust among individuals in a society (Guiso et al., 2004). SC is shaped and theorized through societal relationships (Seibert et al., 2001). People with great SC have a tendency to be further reliable, extra collaborative, and less self-centered (Hasan et al., 2017). In several findings, researchers believe SC is a useful reserve that allows numerous additional sources for instance investment, market knowledge, and clients (Mahfud et al., 2020). Hence, this research analyzes the relationship of social capital with career identity (Ngoma and Dithan Ntale, 2016), job satisfaction (Lange, 2015), and entrepreneurship ability (Light and Dana, 2013). Additionally, this study will analyze the relationships of career identity, job satisfaction, and entrepreneurship ability of ICH bearers with status attainment to discuss in depth the specific relationship between the accumulation of personal capital and the acquisition of the status of ICH bearers.

The social status of ICH bearers should be understood from the interrelationship of various elements of social structure. The status distribution of ICH bearers in the social structure highlights the important aspects of the benign development of traditional culture in China. According to the status attainment theory, the status attainment of ICH bearers is influenced by the “capital stock” they occupy (Rözer and Brashears, 2018). It is the original purpose of this study to enhance the capital stock of ICH bearers, promote their status acquisition, and ultimately realize the sustainable development of ICH. This research covers the following research gaps. First, it explores the relationship of human, psychological, and social capital with career identity, job satisfaction, and entrepreneurship ability. Second, it examines the associations of career identity, job satisfaction, and entrepreneurship ability with status attainment. Third, it analyzes the indirect impacts of human, psychological, and social capital on status attainment. The practical implication of this research is that it broadens the scope for natural transmission, independent cultivation, and intervention of the talent of ICH bearers, thus solving the embarrassing situation of the difficulty of transmission and protection of ICH. It embodies the humanistic care for the ICH bearers and also points out the direction for improving the cultivation of ICH inheritance talents.

## Theoretical Background and Hypotheses Development

### Human Capital and Career Identity

Career identity or occupational identity is an essential notion in the area of career advancement studies. It is strongly associated with a person’s career assessment, career achievement, career decision making, and an individual’s adaptation to the existing complicated occupational situation. Career identity is a relatively clear and stable knowledge of a person’s interests, talents, and goals in his or her occupation, and is the result of an individual’s self-understanding and understanding of the occupational environment (Humlum et al., 2012). The following are some examples of occupational identity Meyers believes that occupational identity is a concept that is gradually constructed and matured by the activities of a particular occupation and that it is through occupational identity that an individual’s interests, abilities, and values are linked to occupational goals (Meijers et al., 2013). Fugate et al. (2004) suggest that an individual’s occupational identity, social and human capital, and psychological capital are all competencies for obtaining career opportunities (Fugate et al., 2004). Gushue’s study found that the higher the individual’s occupational identity, the lower the occupational barriers, as proactive and effective actions increase the likelihood of job opportunities for each individual (Gushue et al., 2006). According to Hall’s theory, occupational identity brings both objective occupational success and positive internal work experiences. Individuals’ occupational success, especially subjective success, is significantly related to occupational identity (Hall and Chandler, 2005). Occupational identity is the core of occupational competence, and occupational competence is an important reflection of occupational status (Holland et al., 1980). Occupational competence is an important reflection of occupational status. Since occupational status is an essential component of status acquisition, this research implies that there is a positive effect between occupational identity and status acquisition. In this research, it is suggested that there is a significant association relationship between the professional identity of ICH representative bearers and their status acquisition.

McArdle proposed that HC is the ability to “know how,” including the amount of education and vocational training and other related skills. Occupational identity, on the other hand, includes occupational motivation and personal values and is the ability to “know why” (McArdle et al., 2007). An individual’s occupational choice is determined by both identity gains and economic gains, and the identity gains are determined by the individual’s personality characteristics, actions, and social type (Akerlof and Kranton, 2002). The benefits of identity are determined by personality characteristics, actions, and social type. Individuals gain economic and non-economic identity gains through human capital enhancement (Humlum et al., 2012). The benefits are determined by the individual’s characteristics, actions, and social type. Skrikoff suggests that education is an essential factor of occupational identity and an important source of improving a person’s work skills, career interests, and career guidance (Vondracek and Skorikov, 1997). Therefore, the improvement of human capital is the most crucial factor in the growth of human resources. Therefore, the improvement of human capital is the basis for the development of occupational identity. Human capital can be used to obtain better employment opportunities through the role of occupational identities such as individual occupational interests and values. Given the above analysis, this study proposes the following hypotheses based on previous studies.

Hypothesis 1: The human capital of ICH bearers has a significant relationship with career identity.

### Human Capital and Job Satisfaction

The extent to which workers are satisfied with their work is vital to corporations because of its connection to turnover and job performance (Van Dick et al., 2004; Hirschi, 2011). Additionally to the elements of Porter and Lawler’s (1968) framework, the characteristics of the HC concept have been applied for the hypothetical framework of this research. HC is explained as the investment training and education of personnel in a company (Wang et al., 2008). People invest in HC, for instance, training, education, discipline, and experience as they anticipate potential gains as outcomes from improved job performance (Langelett, 2002; Lubinski et al., 2006). Job satisfaction (JS) and consequent execution is affected by the efficient matching between HC and anticipated rewards. Workers that stay in a specified career for prolonged times or stay in employment at an organization for a long time are investing in their HC by earning important knowledge and enhancing their abilities (Allen and Van der Velden, 2001; Langelett, 2002). They are instantaneously promoting the overall achievement of the company by assisting the company to achieve the designated job targets (Wang et al., 2008; Lyons and Akroyd, 2014). Human capital can improve individual job satisfaction and job performance. Research data indicate that investment in human capital has a significant impact on the job satisfaction of employees (Jones et al., 2009). In view of the above analysis, this study proposes the following hypotheses based on previous studies.

Hypothesis 2: The human capital of ICH bearers has a significant relationship with job satisfaction.

### Human Capital and Entrepreneurship Ability

The study of entrepreneurship began in the 1990s in the West. Entrepreneurial ability is the ability of social individuals or business organizations to create or accomplish new things (Zahra et al., 2006). The study of entrepreneurial capabilities began in the 1990s. Current research on entrepreneurial capacity is both qualitative and quantitative (Man et al., 2002). There are both qualitative and quantitative approaches to entrepreneurship. Many studies have proved that only the ability to think and analyze and the ability to act can contribute to the success of entrepreneurship (Markman and Baron, 2003; Karlsson and Honig, 2009). Some studies suggest that risk-taking and commitment are the necessary abilities for entrepreneurs to be successful (Markman and Baron, 2003). Some scholars also suggest that entrepreneurs should have the ability to learn continuously to ensure the success of entrepreneurship (Lans et al., 2008). Some scholars suggest that entrepreneurs should have the ability to learn continuously to ensure successful entrepreneurship.

HC enhances the ultimate entrepreneurial performance by acting on the entrepreneurial ability of individuals. In other words, the development of knowledge and skills for individuals can improve the success rate of entrepreneurship. The importance of HC in entrepreneurship research is longstanding (Marvel et al., 2016). HC theory was initially established to examine the importance of teaching and implied individuals have variable understanding and abilities that have financial significance (Schultz, 1961). Mincer (1958) originally considered the theory of HC as a justification for income disparity. Rises in national productivity were unbalanced concerning property, and employment periods, furthermore, investing in HC is perhaps the most important clarification (Schultz, 1961). Becker (1964) constructed on these ideas and devised the notion of investments in HC established on the enormous quantity of data that additional well skilled and competent individuals practically constantly have a tendency to gain higher than others. The concept has been progressively employed in the field of entrepreneurship, constantly connecting HC characteristics to entrepreneurship ability (Unger et al., 2011). Several influential opinions explain the significance of HC in the area of entrepreneurship (Ardichvili et al., 2003). HC is essential to discover and create entrepreneurship prospects (Alvarez and Barney, 2007; Marvel et al., 2016). HC similarly promotes developing prospects by obtaining economic assets and introducing enterprises (Bruns et al., 2008; Dimov, 2010). HC supports the growth of innovative expertise and the concept of benefits for different organizations (Corbett et al., 2007; Bradley et al., 2012). In practical usage, HC is one of the commonly applied selection measures for investors in assessing prospective enterprise performance (Marvel et al., 2016). So far, in HC and entrepreneurship ability research studies, meta-analytic evaluations of HC and business results can be discovered (Unger et al., 2011) and an analysis of entrepreneurship learning on HC skills, actions, and behavior can be observed (Martin et al., 2013). All offer a persuasive indication that HC is important to entrepreneurship (Marvel et al., 2016). In view of the above analysis, this study proposes the following hypotheses based on previous studies.

Hypothesis 3: The human capital of ICH bearers has a significant relationship with entrepreneurship ability.

### Psychological Capital and Career Identity

PC is a specific optimistic psychological state (Luthans and Youssef, 2004), whereas occupational identity is a person’s individual experience and evaluation of his or her occupation, which affects the individual’s commitment, enthusiasm, and satisfaction with the job. The positive effects of psychological capital on occupational identity have been indirectly described from different perspectives in the research literature. PC has an immediate significant impact on individuals, groups, and organizations (Luthans et al., 2006). Psychological capital is a direct gain for individuals, groups, and organizations. Intrinsically, the professional identity of ICH bearers is both an emotional state and a cognitive process, which is inevitably linked to the psychological characteristics of the ICH bearers themselves. ICH bearers are equipped with a complete technical system and are responsible for the mission of ICH transmission; at the same time, they also participate in social activities. At the psychological level, the cognition tends to be mature and the will to be independent is strong. According to positive psychology, the greater the PC of ICH inheritors, the greater their positive occupational identity. They are also more likely to devote more time and energy to their work. They also devote more time and energy to develop their potential, stimulate the joy of ICH transmission, and make positive attributions (Luthans et al., 2005). They will also devote more time and energy to their potential, stimulate the joy of ICH transmission, and actively attribute more creativity and motivation to the ICH transmission business, and actively exert extraordinary willpower and resilience when dealing with difficulties (Luthans et al., 2006). In view of the above analysis, this study proposes the following hypotheses based on previous studies.

Hypothesis 4: The psychological capital of ICH bearers has a significant relationship with occupational identity.

### Psychological Capital and Job Satisfaction

The empirical study by the researchers concluded that the impact of PC on job satisfaction is more incremental than HC and SC (Larson and Luthans, 2006). The impact of PC on job satisfaction is more incremental than that of human and social capital. Employees’ resilience, hope, and optimism have a significant association with their job performance and satisfaction, and the effect is more significant (Luthans and Youssef, 2007). The Sun survey found that positive psychological forces have a significant impact on employees’ job satisfaction and attitudes, resulting in a positive motivational force for the organization (Sun et al., 2012). This results in positive motivation for the organization. In view of the above analysis, this study proposes the following hypotheses based on previous studies. In view of the above analysis, this study proposes the following hypotheses based on previous studies.

Hypothesis 5: The psychological capital of ICH bearers has a significant relationship with job satisfaction.

### Psychological Capital and Entrepreneurship Ability

A motivational and intellectual distinction is the important energy that promotes entrepreneurship abilities in a person. The achievement of an entrepreneurship journey remains in the capability to recognize prospects and take essential measures for conquering the entire barriers encountered in the whole procedure that needs both, the entrepreneur’s motivational and cognitive strength (Fakhri et al., 2012). The psychological structure in a person is usually created throughout the infant phase and is mainly affected by the atmosphere in which an individual is raised. It can be asserted that positively inspired entrepreneurs personally manage to accomplish a larger level of success. Moreover, personal qualities usually affect the entrepreneurship abilities of a person. Consequently, the existence of PC has been discovered to enable the entrepreneurship growth of a person. The fundamental characteristics that describe an entrepreneur comprise the capability to accelerate modernization, the capability to take risks, the capability to run a specific group, and several others (Bolton and Lane, 2012). These qualities allow a business to join in the advanced culture and support the company.

PC of the entrepreneurs their teams can improve interaction and management with the shareholders. The characteristics of resilience, hope, and optimism assist entrepreneurs to maintain contact and management even if they encounter any difficulties and disputes. Furthermore, the characteristic of self-efficacy, at individual or team levels, can allow entrepreneurs to connect and organize positively and pleasantly (Tang, 2020). In view of the above analysis, this study proposes the following hypotheses based on previous studies.

Hypothesis 6: The psychological capital of ICH bearers has a significant relationship with entrepreneurship ability.

### Social Capital and Career Identity

Career identity is characterized as the way individuals describe themselves in the career framework, behaving as an intellectual scope utilized to direct career prospects (Fugate et al., 2004). It comprises a sequence of linked capabilities that consist of the understanding, knowing-why capabilities that answer to the reason of executing a job; the knowing-how capabilities that comprise the methodology to execute a job; and the knowing-whom capabilities that include the details about the individuals involved in executing a job (DeFillippi and Arthur, 1994). Additionally, a study revealed that empowered experts with an improved perception of qualified self have the capability to positively network with others for their job development and growth (Johnson and Neary, 2015). Furthermore, career identity is crucial in defining a person’s self-esteem in the course of unemployment. Individuals aware of their activities, abilities, and skills, have knowledge and directions regarding their prospects. Hence, possessing high self-esteem. Moreover, they also consider it simple to cooperate with others to have ideas regarding the ways to accomplish their objectives. This supports them to create and develop their SC (Shaffer and Zhang, 2002). Additionally, it has been discovered that career identities are produced in social communications. Individuals in a similar career direction have a tendency to partner together, thus communicating opinions on the methods to improve the careers that facilitate them to create their social connections (Ibarra, 2005). Most of the issues are related to societal perspectives and activities (West-Burnham et al., 2007). The phrase public value was utilized to illustrate the change in the notion of the ways parties engage with each other to offer services and develop societal effects appreciated by the society (Leadbeater and Mongon, 2008). Nevertheless, investigations indicate that if individuals exhibit substantial and persistent education, organizations must also assist in the provision of SC by offering networking opportunities with the management, society, and co-workers (Catts and Ozga, 2005). Hence, people having networks of society, organization, and, family, that encourage the regulating characteristics of a job are expected to strengthen the importance of a job for that individual. In this manner working to improve an individual’s probability of being employed. Consequently, it can be implied that job identity fills an exceptionally unique position in SC theory (Kagaari, 2007; Ngoma and Dithan Ntale, 2016). In view of the above analysis, this study proposes the following hypotheses based on previous studies.

Hypothesis 7: The social capital of ICH bearers has a significant relationship with career identity.

### Social Capital and Job Satisfaction

SC is deemed to be an accurate predictor of job satisfaction as compared to various other organizational characteristics (Requena, 2003). Investigators started to turn their interest from the scientific to the societal perspective, discovering the social perspective to be an essential motivator of conduct in companies (Agneessens and Wittek, 2008). This claim is supported by other researchers, that find SC has a significant relationship with subjective welfare (Helliwell and Putnam, 2004). Similarly, researchers in organizational psychology can infer that valuable social relationships can provide a strong resource of satisfaction at a job (Rego et al., 2009). Current findings of job satisfaction attach additional credibility to these statements, stating statistically substantial relationships between trust, values, job satisfaction, and, social capital (Fargher et al., 2008; Lange, 2015). In addition, a significant association between job satisfaction and general life’s mindsets and procedures, for instance, socio-cultural beliefs and standards (Georgellis et al., 2012; Lange, 2015). Hence, it can be inferred that SC is capable of having an impact on job satisfaction (Lange, 2015). In view of the above analysis, this study proposes the following hypotheses based on previous studies.

Hypothesis 8: The social capital of ICH bearers has a significant relationship with job satisfaction.

### Social Capital and Entrepreneurship Ability

The position of SC in entrepreneurship ability has developed as a progressively important subject in corporate research, and the discussion has turned gradually more complicated (Casson and Giusta, 2007). Initial providers to this narrative were satisfied to establish SC and then to describe its formerly unrecognized role to entrepreneurship ability (Allen et al., 2000; Burt, 2003). Corresponding to this traditional viewpoint, SC comprises associations of faith and mutual benefit that are required in social groups (Ostrom and Ahn, 2009; Mustafa and Chen, 2010; Slotte-Kock and Coviello, 2010; Light and Dana, 2013). The role of SC to entrepreneurship ability can be specified as the mobilization of resources via social networks of reciprocity and trust (Galbraith et al., 2007; Nahapiet, 2009; Light and Dana, 2013). Nevertheless, further modern approaches to SC have objected that a lot of SC suppresses entrepreneurship ability because it lowers objectivity, and enforces psychological compliance on entire groups (Aldrich and Kim, 2007; Light and Dana, 2013). In view of the above analysis, this study proposes the following hypotheses based on previous studies.

Hypothesis 9: The social capital of ICH bearers has a significant relationship with entrepreneurship ability.

### Career Identity and Status Attainment

Career identity is a fundamental concept for career advancement and has been a key aim of employment psychology. The education and application of a strong feeling of career identity are considered as an essential progressive job that begins in youth and persists through maturity (Hirschi, 2011). This career-growth notion has likewise earned heightened significance from a modern organizational perception. Fugate et al. (2004) identified career identity as a central element of employability. Several studies have indicated that individuals with a stronger perception of career identity were further effective in career modifications and stated superior status attainment and well-being (Gushue et al., 2006; Diemer and Blustein, 2007; Hirschi, 2011). Gushue’s study found that the higher the individual’s occupational identity, the lower the occupational barriers, as proactive and effective actions increase the likelihood of job opportunities for each individual (Gushue et al., 2006). According to Hall’s theory, occupational identity brings both objective occupational success and positive internal work experiences. Individuals’ occupational success, especially subjective success, is significantly related to occupational identity (Hall and Chandler, 2005). A person with a high level of occupational identity In other words, occupational identity is the core of occupational competence, and occupational competence is an important reflection of occupational status (Holland et al., 1980). Occupational competence is an important reflection of occupational status. Since occupational status is an important component of status acquisition, this study suggests that there is a positive effect between occupational identity and status acquisition. In this study, it is suggested that there is a positive relationship between the professional identity of ICH representative bearers and their status acquisition. In view of the above analysis, this study proposes the following hypotheses based on previous studies.

Hypothesis 10: The career identity of ICH bearers has a significant relationship with status attainment.

### Job Satisfaction and Status Attainment

Hoppock (1935) was the first to propose job satisfaction, which refers to the degree to which a person’s job satisfaction is a subjective perception and emotive reaction to the job environment and other conditions, especially from the difference between the actual and expected income of the job. Currently, there are few studies on individual status acquisition in terms of job satisfaction. Related studies mainly focus on the impact of job satisfaction on employees’ performance (Russ and McNeilly, 1995). The study was conducted by using three-dimensional capital as a citation and job satisfaction as an intermediate variable to explore the factors influencing employee innovation performance. The empirical study concluded that employees’ three-dimensional capital had a significant impact on job satisfaction and job involvement, respectively. The synergistic effect of three-dimensional capital could also affect employees’ innovation performance and job satisfaction (Demir, 2018; Gong et al., 2019). Job performance is an important component of occupational status (Haibo et al., 2018) and occupational status is an important expression of one’s social status, so this study suggests that there is a significant relationship between job satisfaction and status attainment. In view of the above analysis, this study proposes the following hypotheses based on previous studies.

Hypothesis 11: The job satisfaction of ICH bearers has a significant relationship with status attainment.

### Entrepreneurship Ability and Status Attainment

From this, it can be seen that the entrepreneurial ability of an entrepreneur is the focus of research on business success. Man analyzes the impact of entrepreneurial ability on competitiveness from three dimensions: potential, performance, and process (Man et al., 2002). Ahmad and other scholars have further explored the impact of each dimension of entrepreneurial ability on entrepreneurial success based on Man’s model. Ahmad believes that the entrepreneurial competency dimension is extended from the firm to the individual and adds a “personal competency” dimension, suggesting that entrepreneurs need personal competency support and emphasizing the study of personal characteristics (Ahmad et al., 2010). Entrepreneurial success is also a learning process in which entrepreneurs acquire skills and accumulate knowledge (Wang and Chugh, 2014). This suggests that only when entrepreneurs have the technical, organizational, and market development skills can they effectively achieve entrepreneurial success (Ozgen and Minsky, 2006; Yahya et al., 2011; Arend, 2014). This suggests that entrepreneurial success can only be achieved effectively if entrepreneurs have the technical, organizational, and market development skills. The representative heirs of ICH have multiple identities of project representatives and business operators in a certain sense. The safety and conservation of ICH also have the connotation of entrepreneurial development. Since entrepreneurial success is an important expression of occupational status, and occupational status is an important expression of one’s social status, this study concludes that there is a positive relationship between one’s entrepreneurial ability and status acquisition. In view of the above analysis, this study proposes the following hypotheses based on previous studies.

Hypothesis 12: The entrepreneurship ability of ICH bearers has a significant relationship with status attainment.

### Career Identity and Job Satisfaction

Having a certain perception of an individual’s powers and inclinations enables the choice of self-consistent career objectives (Skorikov and Vondracek, 2007; Hirschi et al., 2011). These objectives in turn help goal attainment and accomplishment, which promotes job satisfaction. Likewise, accomplishing self-congruent objectives is additional gratifying than completing extrinsically determined ambitions (Judge et al., 2005; Hirschi, 2011). A certain perception of identity likewise encourages the awareness of relevance in work, which is in turn significantly associated with job satisfaction (Humphrey et al., 2007; Dik and Duffy, 2009). Lastly, a certain idea of identity is associated with job engagement (Luyckx et al., 2010; Hirschi, 2011), which might likewise stimulate the perception of accomplishment regarding job goals and job satisfaction. Consequently, career identity can be anticipated to encourage job satisfaction (Hirschi, 2011). In view of the above analysis, this study proposes the following hypotheses based on previous studies.

Hypothesis 13: The career identity of ICH bearers has a significant relationship with job satisfaction.

### Entrepreneurship Ability and Job Satisfaction

From a traditional financial perspective, the option of being an entrepreneur exhibits a few puzzling characteristics (Hamilton, 2000). It appears to provide high value, for instance, entrepreneurs frequently account greater levels of job satisfaction as compared to employees with comparable attributes (Benz and Frey, 2008; Bianchi, 2012). Economic growth raises benefit disparities among the employees and the self-employed. Furthermore, this impact is described by improved profits and non-monetary attributes of job satisfaction and, in specific, autonomy.

Moreover, the presence of value variations is not because of certain market defects. Hence, It seems that a wider analysis of the way various institutions and markets impact non-monetary gains from a job might be of great interest to researchers (Bianchi, 2012). In view of the above analysis, this study proposes the following hypotheses based on previous studies.

Hypothesis 14: The entrepreneurship ability of ICH bearers has a significant relationship with job satisfaction.

**FIGURE 1:**
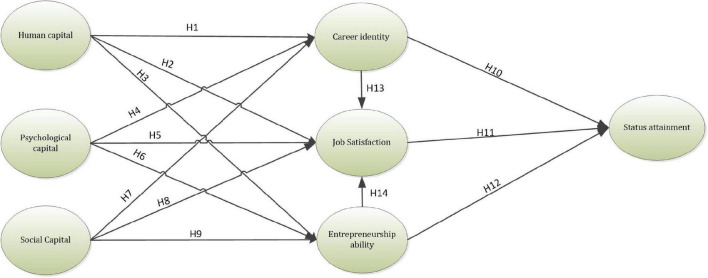
Theoretical framework.

Figure 1 indicates the research model for this study.

## Materials and Methods

This study adopts a research method that combines quantitative and qualitative research. Quantitative methods are used to improve the reliability of research data and findings, while qualitative methods are used to further investigate and explain different aspects of the problem. The interview outline was designed by combining the relevant contents of the literature. Convenience sampling technique was employed to select the samples, through the introduction of instructors, teachers, and close friends. The interview invitation to the interviewees was communicated by telephone and email. Through the interviews, abundant first-hand information was obtained. Furthermore, the feelings and knowledge of experts, scholars, and ICH bearers, were understood and their views and suggestions on the identification of ICH bearers, cultivation and protection, and social status enhancement were also obtained, to lay a solid qualitative research foundation for this study.

A questionnaire survey was conducted to obtain basic information and professional experiences of ICH bearers to provide real support for the research analysis and discussion. To measure and demonstrate the actual needs of various “capital stocks” of different types of ICH bearers, a scale analysis was adopted. The design of the questionnaire was based on previous research, combined with expert interviews and national-level ICH heir recognition standards, and mainly collected basic personal information, social status information, heir experience information, and current industry experience information of ICH heirs. The specific relationship between the capital reserve and the social status of ICH bearers is presented.

In terms of obtaining basic personal information, the questions mainly covered gender, age, origin, current residence, lineage, and occupation. To measure the social status information, the measurement items mainly focused on collecting information on the perceived social status of ICH bearers, specifically measured in three dimensions: occupational prestige, economic income, and social recognition. Regarding the collection of information on bearers’ experiences, the questionnaire questions the experience of the bearer, in terms of the project, rank, and years of experience of the ICH bearer. A total of 600 questionnaires were distributed and collected employing a convenience sampling technique mainly through the help of instructors, referrals from classmates and friends, the help of government cultural departments, and publicity departments. The distribution and collection methods were mainly through on-site visits, e-mail boxes, online and real-time communication software. Finally, there was a total of 506 participants in this study. The sample consists of 61.9% males and 38.1% females. About 65.2% of the respondents are 36–59 years old, 18.4% below 35 and the rest above 60 years old. Besides, 52.6% education level of the samples are high school or less, 41.1% university/college and the rest postgraduate degree.

## Results

### Data Analysis

Two approaches of calculating and estimating partial least squares (PLS) were implemented. In the primary stage, the reliability estimation was conducted. The second phase was related to the descriptive analysis and evaluation of the research model. The aim of the two stages acknowledged previously was to authenticate the construct’s reliability, involving confirming the connection among the constructs (Anderson and Gerbing, 1988; Hulland, 1999). PLS has been employed and believed as the most excellent means for describing the basic collaboration among variables and thus can simultaneously manage framework variables and measurement items (Petter et al., 2007). Additionally, as PLS has simpler restrictions for changing normality and ambiguity; it is ideal for investigating the connection if the constructs are irregularly dispersed. Hence, It possesses the benefit of calculating dynamical research frameworks (Chin et al., 2003). PLS was consequently applicable for this analysis than earlier SEM assessment practices to calculate the relationships between constructs, lessen measurement errors, and avoid collinearity.

### Convergent and Discriminant Validity

The structural equation modeling (SEM) technique is employed to examine the projected hypotheses founded in the aforementioned segment of this research, and as a result, the Smart PLS 3.2.8 was utilized. PLS-SEM procedure is applicable for regular and complex research structures. Similarly, researchers concluded that PLS-SEM is a sustainable practice for measurement as compared to covariance-based SEM. PLS-SEM is regarded fairly in handling and evaluating calculations for mediation effects as compared to regression (Hair et al., 2014). PLS-SEM incorporates the inner as well as the outer models. The outer model was calculated by three estimations, including individual item reliability, discriminant validity, and convergent validity. Corresponding to Table 1, the peak value for factor loading is 0.904. The lowest value for factor loading is 0.638. These values are discovered to be above the 0.50 factor loading threshold value (Hair et al., 2014). Hence, it can be inferred that this research has no concerns regarding individual item reliability. The internal reliability of variables was calculated with the help of composite reliability (CR). The CR value ought to be greater than the threshold value of 0.60 (Hair et al., 2014). Corresponding to the CR values of this research indicated in Table 1, the internal consistency of each construct was achieved (Bagozzi et al., 1991). This study indicates that the internal consistency requirement is fulfilled. Convergent validity suggests the level of similarity between the item and the relative construct. Corresponding to the conclusions revealed in Table 1, 0.725 is discovered as the greatest value of average variance extracted (AVE). On the other hand, 0.566 is discovered to be the lowest value of AVE. Subsequently, the AVE values of this research are greater than the threshold value of 0.50 and hence, this study assures convergent validity requirement (Hair et al., 2014). Furthermore, Table 1, indicates that all Cronbach alpha values are higher than the threshold value of 0.6 (Taber, 2018).

**TABLE 1:** Construct validity and reliability.

Constructs	Indicators	Factor loadings	Cronbach’s alpha	Composite reliability	Average variance extracted (AVE)
EA	EA1 EA2 EA3 EA4	0.809 0.863 0.868 0.822	0.861	0.906	0.707
HC	HC1 HC2 HC3 HC4 HC5 HC6 HC7	0.766 0.699 0.693 0.766 0.781 0.795 0.733	0.799	0.865	0.566
JS	JS1 JS2 JS3 JS4	0.773 0.866 0.890 0.872	0.872	0.913	0.725
PC	PC1 PC2 PC3 PC4 PC5 PC6 PC7 PC8	0.762 0.761 0.793 0.803 0.778 0.809 0.747 0.785	0.908	0.926	0.608
CI	CI1 CI2 CI3 CI4	0.871 0.904 0.814 0.763	0.859	0.905	0.705
SC	SC1 SC2 SC3 SC4 SC6 SC7 SC8 SC9 SC10 SC12 SC13 SC14 SC15	0.608 0.638 0.638 0.638 0.688 0.728 0.718 0.659 0.715 0.687 0.671 0.662 0.688	0.675	0.825	0.624
SA	SA1 SA2 SA3 SA4 SA5 SA6 SA7 SA8	0.719 0.800 0.797 0.737 0.694 0.779 0.740 0.792	0.894	0.915	0.575

*HC, Human capital; SC, Social capital; PC, Psychological capital; CI, Career identity; JS, Job satisfaction; EA, Entrepreneurship ability; SA, Status attainment.*

The degree of discrimination among analyzing variables and various construct measures is characterized by discriminatory validity. Table 2 implies a decent discriminant validity for every construct, by indicating that the factor loading value of every item is the highest in the latent structure, as compared to other structures. The highest factor loading values are highlighted in yellow (Hair et al., 2016).

**TABLE 2:** Cross loadings.

	EA	HC	JS	PC	CI	SC	SA
EA1	0.809	0.459	0.523	0.544	0.473	0.513	0.533
EA2	0.863	0.471	0.553	0.597	0.476	0.622	0.614
EA3	0.868	0.483	0.571	0.644	0.472	0.602	0.574
EA4	0.822	0.459	0.511	0.563	0.457	0.528	0.543
HC1	0.424	0.766	0.439	0.529	0.428	0.443	0.391
HC2	0.422	0.699	0.376	0.458	0.394	0.397	0.389
HC3	0.334	0.693	0.312	0.397	0.335	0.376	0.316
HC4	0.362	0.766	0.398	0.438	0.368	0.388	0.273
HC5	0.450	0.781	0.411	0.498	0.367	0.471	0.368
HC6	0.388	0.795	0.427	0.493	0.403	0.476	0.336
HC7	0.319	0.733	0.390	0.436	0.388	0.407	0.299
JS1	0.597	0.372	0.773	0.552	0.525	0.558	0.557
JS2	0.488	0.462	0.866	0.598	0.700	0.473	0.429
JS3	0.539	0.469	0.890	0.631	0.702	0.515	0.508
JS4	0.564	0.523	0.872	0.636	0.706	0.524	0.531
PC1	0.527	0.539	0.568	0.762	0.517	0.547	0.484
PC2	0.457	0.544	0.506	0.761	0.530	0.564	0.493
PC3	0.597	0.496	0.530	0.793	0.469	0.596	0.493
PC4	0.559	0.528	0.641	0.803	0.588	0.554	0.478
PC5	0.569	0.511	0.480	0.778	0.434	0.605	0.543
PC6	0.594	0.474	0.545	0.809	0.447	0.543	0.510
PC7	0.514	0.433	0.489	0.747	0.444	0.547	0.538
PC8	0.546	0.472	0.647	0.785	0.589	0.570	0.532
CI1	0.464	0.443	0.659	0.544	0.871	0.410	0.402
CI2	0.472	0.458	0.692	0.561	0.904	0.424	0.398
CI3	0.483	0.441	0.651	0.557	0.814	0.460	0.402
CI4	0.454	0.394	0.599	0.512	0.763	0.453	0.453
SC1	0.287	0.246	0.241	0.333	0.197	0.608	0.404
SC2	0.339	0.333	0.309	0.386	0.251	0.638	0.385
SC3	0.329	0.307	0.272	0.362	0.199	0.638	0.402
SC4	0.506	0.313	0.344	0.402	0.269	0.638	0.571
SC6	0.380	0.322	0.390	0.427	0.324	0.688	0.478
SC7	0.406	0.429	0.423	0.467	0.357	0.728	0.469
SC8	0.406	0.353	0.350	0.443	0.274	0.718	0.487
SC9	0.475	0.312	0.384	0.410	0.305	0.659	0.529
SC10	0.503	0.525	0.502	0.598	0.417	0.715	0.449
SC12	0.478	0.460	0.483	0.541	0.392	0.687	0.442
SC13	0.569	0.433	0.464	0.551	0.427	0.671	0.542
SC14	0.558	0.472	0.491	0.587	0.490	0.662	0.534
SC15	0.537	0.548	0.477	0.634	0.427	0.688	0.483
SA1	0.505	0.404	0.545	0.576	0.461	0.564	0.719
SA2	0.528	0.418	0.522	0.573	0.465	0.595	0.800
SA3	0.555	0.353	0.462	0.516	0.357	0.558	0.797
SA4	0.508	0.439	0.553	0.564	0.480	0.533	0.737
SA5	0.421	0.294	0.309	0.337	0.259	0.426	0.694
SA6	0.539	0.358	0.426	0.445	0.313	0.562	0.779
SA7	0.442	0.392	0.383	0.443	0.334	0.462	0.740
SA8	0.565	0.360	0.355	0.445	0.274	0.517	0.792

*HC, Human capital; SC, Social capital; PC, Psychological capital; CI, Career identity; JS, Job satisfaction; EA, Entrepreneurship ability; SA, Status attainment. The highest factor loading of each item in the latent structure is highlighted in yellow.*

Tenenhaus et al.’s (2005) equation was employed to calculate the goodness of fit (GOF) for this study. The quality of the research framework is analyzed as follows:


G⁢O⁢F=A⁢V⁢E¯⁢x⁢R2¯=0.644⁢x⁢ 0.544=0.60

Subsequent to the aforementioned result, the GOF is 0.60 that attains the 0.40 cut-off requirements for a substantial impact size (Wetzels et al., 2009).

### Structural Model Analysis

The path analysis of this research was estimated by employing Smart PLS 3.2.8. The inner model was analyzed and according to researchers, the *p*-value should be lower than the threshold value of 0.05. On the other hand, the *t*-value should be higher than the threshold value of 1.96.

**FIGURE 2:**
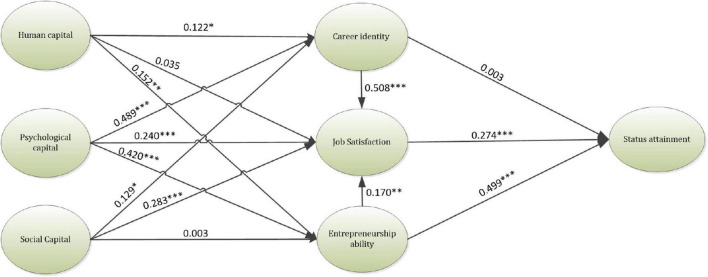
Results of the inner model. ^***^p-value < 0.001, ^**^p-value < 0.01, *p-value < 0.05.

As per the results of this research, as shown in Table 3 and Figure 2. HC was found to be in a significant relationship with career identity and entrepreneurship ability, hence, H1 (β = 0.112, *t*-value = 2.042) and H3 (β = 0.152, *t*-value = 2.874) were supported. Moreover, PC was discovered to significantly influence career identity H4 (β = 0.489, *t*-value = 7.940), job satisfaction H5 (β = 0.240, *t*-value = 3.926), and, entrepreneurship ability H6 (β = 0.420, *t*-value = 6.779). Additionally, SC significantly impacted career identity, and entrepreneurship ability, consequently supporting H7 (β = 0.129, *t*-value = 2.267) and H8 (β = 0.283, *t*-value = 4.649). Furthermore, JS and EA were found to be in a significant relationship with status attainment, thereby supporting H11 (β = 0.274, *t*-value = 4.742), and H12 (β = 0.449, *t*-value = 10.955). In addition, career identity and EA were discovered to significantly impact JS, hence H13 (β = 0.508, *t*-value = 10.845) and H14 (β = 0.170, *t*-value = 3.097) were accepted. Consequently, all the hypotheses in this research were supported except H2, H9, and H10. According to the results, HC did not have any significant impact on JS, hence H2 (β = 0.035, *t*-value = 0.696) was rejected. Furthermore, SC was not in a significant association with career identity, therefore H9 (β = 0.003, *t*-value = 0.0076) was not supported. Moreover, career identity and status attainment did not have a significant relationship, consequently H10 (β = 0.003, *t*-value = 0.065) was rejected.

**TABLE 3:** Hypothesis results.

Hypothesis	Path coefficients (β)	*T*-values	*P*-values	Results
H1: HC - > CI	0.122	2.042	0.041	Supported
H2: HC - > JS	0.035	0.696	0.486	Not supported
H3: HC - > EA	0.152	2.874	0.004	Supported
H4: PC - > CI	0.489	7.940	0.000	Supported
H5: PC - > JS	0.240	3.926	0.000	Supported
H6: PC - > EA	0.420	6.779	0.000	Supported
H7: SC - > CI	0.129	2.267	0.023	Supported
H8: SC - > EA	0.283	4.649	0.000	Supported
H9: SC - > JS	0.003	0.076	0.940	Not supported
H10: CI - > SA	0.003	0.065	0.948	Not supported
H11: JS - > SA	0.274	4.742	0.000	Supported
H12: EA - > SA	0.499	10.955	0.000	Supported
H13: CI - > JS	0.508	10.845	0.000	Supported
H14: EA - > JS	0.170	3.097	0.002	supported

*HC, Human capital; SC, Social capital; PC, Psychological capital; CI, Career identity; JS, Job satisfaction; EA, Entrepreneurship ability; SA, Status attainment.*

This study utilized, the outcomes of the indirect effects by Smart PLS as specified in Table 4. Corresponding to the outcomes indicated in Table 4, Human capital was found to be in a significant indirect relationship with status attainment via entrepreneurship ability. Furthermore, PC was found to be in a significant indirect association with status attainment, with job satisfaction and entrepreneurship ability as mediator variables. Lastly, social capital was in an indirect relationship with status attainment, via entrepreneurship ability as a mediator.

**TABLE 4:** Indirect effects result.

	Path coefficients (β)	*T*-values	*P*-values	Results
HC - > CI - > SA	0.000	0.056	0.955	Not supported
HC - > JS - > SA	0.009	0.684	0.494	Not supported
HC - > EA - > SA	0.076	2.758	0.006	Supported
PC - > CI - > SA	0.001	0.064	0.949	Not supported
PC - > JS - > SA	0.066	2.965	0.003	Supported
PC - > EA - > SA	0.209	6.009	0.000	Supported
SC - > CI - > SA	0.001	0.061	0.952	Not supported
SC - > EA - > SA	0.142	3.959	0.000	Supported
SC - > JS - > SA	0.002	0.073	0.942	Not supported

*HC, Human capital; SC, Social capital; PC, Psychological capital; CI, Career identity; JS, Job satisfaction; EA, Entrepreneurship ability; SA, Status attainment.*

## Conclusion and Discussion

Based on the status acquisition model, this study analyzed the HC, SC, and PC of ICH bearers from three perspectives. In addition to the conventional socio-demographic factors such as gender, age, place of residence, and education level as control variables, status attainment was introduced as a dependent variable, and occupational identity, job satisfaction, and entrepreneurial ability were introduced as mediating variables to construct a conceptual model in the hope of exploring the multiple influencing factors of status attainment among ICH bearers. This study attempts to apply the basic framework of “three-dimensional capital theory” to the field of sociological research to analyze the transmission and protection of ICH, and to discuss in depth the specific relationship between the accumulation of personal capital and the acquisition of the status of ICH bearers.

As per the results of this research, all the hypotheses were supported except H2, H9, and H10. Corresponding to the findings of this research, human capital did not have any significant impact on job satisfaction, this result was somewhat different from a research survey conducted by Lyons and Akroyd (2014) in the community college of United States, according to their results HC was found to be in a significant relationship with job satisfaction and job performance. Additionally, the results of this study indicated an insignificant association between SC and career identity. This finding tends to explain that social networks are not necessarily important to impact individuals feeling toward jobs. Furthermore, career identity and status attainment did not have a significant relationship. The results were not in accordance with a previous study conducted by Zhengde et al. (2017).

The practical significance of this study is that it broadens the scope for natural transmission, independent cultivation, and intervention of the talent of ICH bearers, thus solving the embarrassing situation of the difficulty of transmission and protection of ICH. It embodies the humanistic care for the ICH bearers and also points out the direction for improving the cultivation of ICH inheritance talents.

### Theoretical Implications

The theoretical significance of this study is to expand the theoretical “space” for the analysis of the transmission development of ICH bearers and to expand the interpretation and application of the human capital theory (Blaug, 1976), social capital theory (Seibert et al., 2001), psychological capital theory (Luthans et al., 2004), and the status acquisition theory (Rözer and Brashears, 2018). Inspired by the sociologist Merton, this study attempts to break away from the monolithic or absolutist application of the paradigm. The study attempts to break away from the monolithic or absolute application of paradigms. The study attempts to study the status acquisition of ICH bearers from multiple perspectives, theories, and disciplines, including social capital in sociology, human capital in management, and psychological capital in psychology, to provide broader research space and a diversified theoretical basis for the study of the protection, transmission, and development of ICH in China.

In the face of the rich ICH resources, multi-level transmission subjects, and changes in the ecological environment of ICH caused by the modernization of urbanization in China, the “identification and protection of ICH transmission subjects has exceeded the capacity of solving the problem in concrete practice.” This means that the general model temporary measures are no longer able to cope with the changing characteristics of ICH protection, and the unidirectional governmental policy interventions cannot meet the practical needs of the sustainable development of ICH. It is urgent to consider the issues of ICH protection, transmission, and development from multiple disciplines and perspectives, such as management, psychology, and sociology, and to expand the theoretical space for the analysis of the transmission and development of ICH inheritors.

It is not difficult to find that the research on ICH inheritance has been crossed by multiple disciplines and perspectives, and more studies have proved that in the face of specific problems in practice, a single government intervention policy is bound to face “malfunction” or “lack of strength of mind.” “The reason is that the development of diverse ICH resources has led to a lag in the formulation of government policies and a limited scope of universal applicability.” On the one hand, this is reflected in a single policy implementation force and a lack of social participation, which in general presents a “dragging forward” model in which the government is dominant. On the other hand, the single policy recognition standard ignores the actual problem of unreasonable talent structure of ICH inheritance, and the policy orientation of recognizing representative inheritors of ICH is based on a social group perspective, which has limited universality (Wei et al., 2021). The problem with this group perspective is its limited universality.

This study attempts to analyze the relationship between the stock accumulation of ICH bearers and their social status acquisition in the process of inheritance and protection from the perspective of capital, and to describe the social class distribution of ICH bearers in the traditional art category, and to analyze the relationship between the different stocks of the capital of ICH bearers and their social status acquisition in depth. To find intervention strategies to enhance the capital of ICH bearers, we will also provide an in-depth analysis of the relationship between the capital stock of ICH bearers and their social status, and then find intervention strategies to enhance the capital of ICH bearers. In the end, we will promote the protection, transmission, and development of ICH.

### Practical Implications

The practical significance of this study is to enhance the targeted interventions in the capital stock process of ICH bearers. It is based on the talent cultivation of ICH bearers in the process of inheritance and transmission and seeks to improve the targeting of ICH bearers in the recognition of representative inheritance status, and then improve the status of ICH bearers to obtain, which is also the most direct practical point of this study.

In the analysis of previous studies, it can be seen that the existing policy formulation and incentive mechanisms are more inclined to protect and help the recognized representative bearers of ICH, such as the program “Inheritors,” which uses new media to promote ICH and raises the cultural consciousness of the public to protect ICH by showcasing the performances of the bearers of ICH items and their teams; and the “Beijing Intangible Cultural Heritage The Beijing Municipal Intangible Cultural Heritage Protection Special Fund Management Measures support ICH transmission and protection in three ways: subsidies, interest subsidies and incentives; the Beijing Municipal Government also supports ICH transmission activities by purchasing the works of representative ICH bearers.” These interventions are only targeted at the established ICH representative bearers, and the emphasis on the individual differences of ICH heritage groups is weak. As a result, ICH representative bearers are not able to fully utilize their strengths when they are identified, which leads to a waste of resources and even makes it difficult for ICH projects to be protected and passed on. It is of great practical significance to analyze the relationship between the social status of ICH representative bearers and the accumulated stock of three-dimensional capital and to intervene in the three-dimensional capital of ICH bearers in the long process of ICH transmission.

Currently, most of the existing research perspectives focus on the identification of ICH representative bearers or the problems in the development of ICH heritage as a recognized representative bearer, and tend to explore the “results” of the identification of ICH representative bearers, but rarely from the perspective of the cultivation of ICH bearers. In this study, we attempted to examine the “results” of the recognition of ICH bearers, but rarely from the perspective of intervention in the process of cultivating ICH bearers. To this end, this study attempts to break through the issues of identification and status acquisition of ICH representative bearers in a targeted manner, starting from the identification process of ICH bearers’ cultivation and the accumulation of three kinds of capital stocks of ICH bearers. At present, the development of ICH in China has the advantage of abundant ICH resources, which can meet the needs of China’s cultural development in a short period of time, but in the end, it is difficult to cope with the impact and challenges brought by the rapid development of cultural consumption in the new era on the transmission and development of ICH.

The most important practical significance of this study is discussing the status acquisition of ICH bearers is that through the analysis of the status acquisition of ICH bearers in the social structure, we can provide a targeted solution to enhance the capital reserve, improve the competitiveness of ICH representative bearers in recognition and status acquisition, and solve the fundamental problem of cultivating bearers for the sustainable development of ICH in China. It also provides a solution for the sustainable development of ICH in China and provides effective assistance for the sustainable development of ICH projects.

### Limitations and Future Research Directions

This research has several limitations and future directions. First, the investigation sample is from China, which is an emerging economy. Thus, the findings of the research might not essentially be generalizable to developed economies. Furthermore, people in China follow Confucian culture hence they tend to conform with the masses. Consequently, future researchers can conduct this research study in different economies following various societal norms to get new insights regarding the ICH barriers. Additionally, several other considerations affect ICH bearers’ attitudes. Hence, future investigators can add necessary constructs based on theory of planned behavior (TPB) theories to the research framework to offer a better explanation. Finally, this research can be conducted in the future as follow-up research and analyze the latest situation of ICH (Wei et al., 2021).

## Data Availability Statement

The raw data supporting the conclusions of this article will be made available by the authors, without undue reservation.

## Author Contributions

JZ, CW, and ZW: conceptualization and methodology, and writing about review and editing. JZ: formal analysis, investigation, and visualization. ZW: validation. JZ, LH, CW, ZW, YR, ZH, SZ, and LZ: writing about original draft preparation. All authors have read and agreed to the published version of the manuscript.

## Conflict of Interest

The authors declare that the research was conducted in the absence of any commercial or financial relationships that could be construed as a potential conflict of interest.

## Publisher’s Note

All claims expressed in this article are solely those of the authors and do not necessarily represent those of their affiliated organizations, or those of the publisher, the editors and the reviewers. Any product that may be evaluated in this article, or claim that may be made by its manufacturer, is not guaranteed or endorsed by the publisher.

## References

[B1] Agarwal N. C. (1981). Determinants of executive compensation. *Ind. Relat.* 20 36–45. 10.1111/j.1468-232X.1981.tb00180.x

[B2] Agneessens F. Wittek R. (2008). Social capital and employee well-being: disentangling intrapersonal and interpersonal selection and influence mechanisms. *Rev. Fr. Sociol.* 49 613–637. 10.3917/rfs.493.0613 18052372

[B3] Ahmad N. H. Halim H. A. Zainal S. R. M. (2010). Is entrepreneurial competency the silver bullet for SME success in a developing nation. *Int. Bus. Manage.* 4 67–75. 10.3923/ibm.2010.67.75

[B4] Akerlof G. A. Kranton R. E. (2002). Identity and schooling: some lessons for the economics of education. *J. Econ. Lit.* 40 1167–1201. 10.1257/.40.4.1167

[B5] Aldrich H. E. Kim P. H. (2007). Small worlds, infinite possibilities? How social networks affect entrepreneurial team formation and search. *Strateg. Entrep. J.* 1 147–165. 10.1002/sej.8

[B6] Allen J. Van der Velden R. (2001). Educational mismatches versus skill mismatches: effects on wages, job satisfaction, and on-the-job search. *Oxf. Econ. Papers* 53 434–452. 10.1093/oep/53.3.434

[B7] Allen T. D. Herst D. E. Bruck C. S. Sutton M. (2000). Consequences associated with work-to-family conflict: a review and agenda for future research. *J. Occup. Health Psychol.* 5 278–308. 10.1037/1076-8998.5.2.278 10784291

[B8] Alvarez S. A. Barney J. B. (2007). Discovery and creation: alternative theories of entrepreneurial action. *Strateg. Entrep. J.* 1 11–26. 10.1002/sej.4

[B9] Anderson J. C. Gerbing D. W. (1988). Structural equation modeling in practice: a review and recommended two-step approach. *Psychol. Bull.* 103 411–423. 10.1037/0033-2909.103.3.411

[B10] Ardichvili A. Cardozo R. Ray S. (2003). A theory of entrepreneurial opportunity identification and development. *J. Bus. Ventur.* 18 105–123. 10.1016/S0883-9026(01)00068-4

[B11] Arend R. J. (2014). Entrepreneurship and dynamic capabilities: how firm age and size affect the ‘capability enhancement–SME performance’ relationship. *Small Bus. Econ.* 42 33–57. 10.1007/s11187-012-9461-9 10.1007/s11187-012-9461-9

[B12] Bagozzi R. P. Yi Y. Phillips L. W. (1991). Assessing construct validity in organizational research. *Adm. Sci. Q.* 36 421–458. 10.2307/2393203

[B13] Ballout H. I. (2007). Career success: the effects of human capital, person-environment fit and organizational support. *J. Manage. Psychol.* 22 741–765. 10.1108/02683940710837705

[B14] Becker W. C. (1964). Consequences of different kinds of parental discipline. *Rev. Child Dev. Res.* 1 169–208.

[B15] Benz M. Frey B. S. (2008). The value of doing what you like: evidence from the self-employed in 23 countries. *J. Econ. Behav. Organ.* 68 445–455. 10.1016/j.jebo.2006.10.014

[B16] Bianchi M. (2012). Financial development, entrepreneurship, and job satisfaction. *Rev. Econ. Stat.* 94 273–286. 10.1162/REST_a_00156

[B17] Blaug M. (1976). The empirical status of human capital theory: a slightly jaundiced survey. *J. Econ. Lit.* 14 827–855.

[B18] Bolton D. L. Lane M. D. (2012). Individual entrepreneurial orientation: development of a measurement instrument. *Educ. Train.* 54 219–233. 10.1108/00400911211210314

[B19] Bradley S. W. McMullen J. S. Artz K. Simiyu E. M. (2012). Capital is not enough: innovation in developing economies. *J. Manage. Stud.* 49 684–717. 10.1111/j.1467-6486.2012.01043.x

[B20] Bruns V. Holland D. V. Shepherd D. A. Wiklund J. (2008). The role of human capital in loan officers’ decision policies. *Entrep. Theory Pract.* 32 485–506. 10.1111/j.1540-6520.2008.00237.x

[B21] Burt R. S. (2003). The social structure of competition. *Netw. Knowl. Econ.* 13 57–91. 10.1093/oso/9780195159509.003.0006 33782627

[B22] Casson M. Giusta M. D. (2007). Entrepreneurship and social capital: analysing the impact of social networks on entrepreneurial activity from a rational action perspective. *Int. Small Bus. J.* 25 220–244. 10.1177/0266242607076524

[B23] Catts R. Ozga J. (2005). *What is Social Capital and How Might it be Used in Scotland’s Schools?.* Edinburgh: Centre for Educational Sociology.

[B24] Chin W. W. Marcolin B. L. Newsted P. R. (2003). A partial least squares latent variable modeling approach for measuring interaction effects: results from a Monte Carlo simulation study and an electronic-mail emotion/adoption study. *Inf. Syst. Res.* 14 189–217. 10.1287/isre.14.2.189.16018 19642375

[B25] Corbett A. C. Neck H. M. DeTienne D. R. (2007). How corporate entrepreneurs learn from fledgling innovation initiatives: cognition and the development of a termination script. *Entrep. Theory Pract.* 31 829–852. 10.1111/j.1540-6520.2007.00208.x

[B26] DeFillippi R. J. Arthur M. B. (1994). The boundaryless career: a competency-based perspective. *J. Organ. Behav.* 15 307–324. 10.1002/job.4030150403

[B27] Demir S. (2018). The relationship between psychological capital and stress, anxiety, burnout, job satisfaction, and job involvement. *Eurasian J. Educ. Res.* 18 137–154.

[B28] Diemer M. A. Blustein D. L. (2007). Vocational hope and vocational identity: urban adolescents’ career development. *J. Career Assess.* 15 98–118. 10.1177/1069072706294528

[B29] Dik B. J. Duffy R. D. (2009). Calling and vocation at work: definitions and prospects for research and practice. *Couns. Psychol.* 37 424–450. 10.1177/0011000008316430

[B30] Dimov D. (2010). Nascent entrepreneurs and venture emergence: opportunity confidence, human capital, and early planning. *J. Manage. Stud.* 47 1123–1153. 10.1111/j.1467-6486.2009.00874.x

[B31] Fakhri K. P. Ghanimat P. Koopahi M. Behnie S. (2012). The study of the effects of personality and psychological traits approach on the rate of entrepreneurship. *J. Basic Appl. Sci. Res.* 2 4159–4166.

[B32] Fargher S. Kesting S. Lange T. Pacheco G. (2008). Cultural heritage and job satisfaction in Eastern and Western Europe. *Int. J. Manpow.* 29 630–650. 10.1108/01437720810908938

[B33] Fugate M. Kinicki A. J. Ashforth B. E. (2004). Employability: a psycho-social construct, its dimensions, and applications. *J. Vocat. Behav.* 65 14–38. 10.1016/j.jvb.2003.10.005

[B34] Galbraith C. S. Rodriguez C. L. Stiles C. H. (2007). Social capital as a club good: the case of ethnic communities and entrepreneurship. *J. Enterpr. Communities* 1 38–53. 10.1108/17506200710736258

[B35] Georgellis Y. Lange T. Tabvuma V. (2012). The impact of life events on job satisfaction. *J. Vocat. Behav.* 80 464–473. 10.1016/j.jvb.2011.12.005

[B36] Gong Z. Chen Y. Wang Y. (2019). The influence of emotional intelligence on job burnout and job performance: mediating effect of psychological capital. *Front. Psychol.* 10:2707. 10.3389/fpsyg.2019.02707 31920783 PMC6916327

[B37] Guiso L. Sapienza P. Zingales L. (2004). The role of social capital in financial development. *Am. Econ. Rev.* 94 526–556. 10.1257/0002828041464498

[B38] Gushue G. V. Scanlan K. R. Pantzer K. M. Clarke C. P. (2006). The relationship of career decision-making self-efficacy, vocational identity, and career exploration behavior in African American high school students. *J. Career Dev.* 33 19–28. 10.1177/0894845305283004

[B39] Haibo Y. Xiaoyu G. Xiaoming Z. Zhijin H. (2018). Career adaptability with or without career identity: how career adaptability leads to organizational success and individual career success? *J. Career Assess.* 26 717–731. 10.1177/1069072717727454

[B40] Hair J. F. Jr. Hult G. T. M. Ringle C. Sarstedt M. (2016). *A Primer on Partial Least Squares Structural Equation Modeling (PLS-SEM).* Thousand Oaks, CA: Sage publications.

[B41] Hair J. F. Jr. Sarstedt M. Hopkins L. Kuppelwieser V. G. (2014). Partial least squares structural equation modeling (PLS-SEM): an emerging tool in business research. *Eur. Bus. Rev.* 26 106–121. 10.1108/EBR-10-2013-0128

[B42] Hall D. T. Chandler D. E. (2005). Psychological success: when the career is a calling. *J. Organ. Behav.* 26 155–176. 10.1002/job.301

[B43] Hamilton B. H. (2000). Does entrepreneurship pay? An empirical analysis of the returns to self-employment. *J. Polit. Econ.* 108 604–631. 10.1086/262131

[B44] Hasan I. Hoi C. K. Wu Q. Zhang H. (2017). Does social capital matter in corporate decisions? Evidence from corporate tax avoidance. *J. Account. Res.* 55 629–668. 10.1111/1475-679X.12159

[B45] Helliwell J. F. Putnam R. D. (2004). The social context of well–being. *Philos. Trans. R. Soc. Lond. Ser. B Biol. Sci.* 359 1435–1446. 10.1098/rstb.2004.1522 15347534 PMC1693420

[B46] Hirschi A. (2011). Vocational identity as a mediator of the relationship between core self-evaluations and life and job satisfaction. *Appl. Psychol.* 60 622–644. 10.1111/j.1464-0597.2011.00450.x

[B47] Hirschi A. Niles S. G. Akos P. (2011). Engagement in adolescent career preparation: social support, personality and the development of choice decidedness and congruence. *J. Adolesc.* 34 173–182. 10.1016/j.adolescence.2009.12.009 20074789

[B48] Holland J. J. Gottfredson D. C. Power P. G. (1980). Some diagnostic scales for research in decision making and personality: identity, information, and barriers. *J. Pers. Soc. Psychol.* 39 1191–1200. 10.1037/h0077731

[B49] Hoppock R. (1935). *Job Satisfaction.* Oxford: Harper.

[B50] Hulland J. (1999). Use of partial least squares (PLS) in strategic management research: a review of four recent studies. *Strateg. Manage. J.* 20 195–204. 10.1002/(SICI)1097-0266(199902)20:2<195::AID-SMJ13>3.0.CO;2-7

[B51] Humlum M. K. Kleinjans K. J. Nielsen H. S. (2012). An economic analysis of identity and career choice. *Econ. Inq.* 50 39–61. 10.1111/j.1465-7295.2009.00234.x

[B52] Humphrey S. E. Nahrgang J. D. Morgeson F. P. (2007). Integrating motivational, social, and contextual work design features: a meta-analytic summary and theoretical extension of the work design literature. *J. Appl. Psychol.* 92 1332–1356. 10.1037/0021-9010.92.5.1332 17845089

[B53] Ibarra H. (2005). *Identity Transitions: Possible Selves, Liminality and the Dynamics of Career Change.* Fontainbleau: INSEAD.

[B54] Johnson C. Neary S. (2015). Enhancing professionalism–progressing the career development sector. *J. Natl. Inst. Career Educ. Couns.* 35 57–62.

[B55] Jones M. K. Jones R. J. Latreille P. L. Sloane P. J. (2009). Training, job satisfaction, and workplace performance in Britain: evidence from WERS 2004. *Labour* 23 139–175. 10.1111/j.1467-9914.2008.00434.x

[B56] Judge T. A. Bono J. E. Erez A. Locke E. A. (2005). Core self-evaluations and job and life satisfaction: the role of self-concordance and goal attainment. *J. Appl. Psychol.* 90 257–268. 10.1037/0021-9010.90.2.257 15769236

[B57] Kagaari J. R. (2007). Evaluation of the effects of vocational choice and practical training on students’ employability. *J. Eur. Ind. Train.* 31 449–471. 10.1108/03090590710772640

[B58] Karlsson T. Honig B. (2009). Judging a business by its cover: an institutional perspective on new ventures and the business plan. *J. Bus. Ventur.* 24 27–45. 10.1016/j.jbusvent.2007.10.003

[B59] Lange T. (2015). Social capital and job satisfaction: the case of Europe in times of economic crisis. *Eur. J. Ind. Relat.* 21 275–290. 10.1177/0959680114542907

[B60] Langelett G. (2002). Human capital: a summary of the 20th century research. *J. Educ. Finance* 28 1–23.

[B61] Lans T. Hulsink W. Baert H. Mulder M. (2008). Entrepreneurship education in a small business context: insights from the competence-based approach. *J. Enterp. Cult.* 16 363–383. 10.1142/S0218495808000193

[B62] Larson M. Luthans F. (2006). Potential added value of psychological capital in predicting work attitudes. *J. Leadersh. Organ. Stud.* 13 75–92. 10.1177/10717919070130020601

[B63] Leadbeater C. Mongon D. (2008). *Leadership for Public Value: Achieving Valuable Outcomes for Children, Families and Communities.* Nottingham: National College for School Leadership.

[B64] Lenzerini F. (2011). Intangible cultural heritage: the living culture of peoples. *Eur. J. Int. Law* 22 101–120. 10.1093/ejil/chr006

[B65] Light I. Dana L. P. (2013). Boundaries of social capital in entrepreneurship. *Entrep. Theory Pract.* 37 603–624. 10.1111/etap.12016

[B66] Lubinski D. Benbow C. P. Webb R. M. Bleske-Rechek A. (2006). Tracking exceptional human capital over two decades. *Psychol. Sci.* 17 194–199. 10.1111/j.1467-9280.2006.01685.x 16507058

[B67] Luthans F. Avey J. B. Avolio B. J. Norman S. M. Combs G. M. (2006). Psychological capital development: toward a micro-intervention. *J. Organ. Behav.* 27 387–393. 10.1002/job.373

[B68] Luthans F. Avolio B. J. Walumbwa F. O. Li W. (2005). The psychological capital of Chinese workers: exploring the relationship with performance. *Manage. Organ. Rev.* 1 249–271. 10.1111/j.1740-8784.2005.00011.x

[B69] Luthans F. Luthans K. W. Luthans B. C. (2004). Positive psychological capital: beyond human and social capital. *Bus. Horiz.* 47 45–50. 10.1016/j.bushor.2003.11.007

[B70] Luthans F. Youssef C. M. (2004). Human, social, and now positive psychological capital management: investing in people for competitive advantage. *Organ. Dyn.* 33 143–160. 10.1016/j.orgdyn.2004.01.003

[B71] Luthans F. Youssef C. M. (2007). Emerging positive organizational behavior. *J. Manag.* 33 321–349. 10.1177/0149206307300814

[B72] Luyckx K. Duriez B. Klimstra T. A. De Witte H. (2010). Identity statuses in young adult employees: prospective relations with work engagement and burnout. *J. Vocat. Behav.* 77 339–349. 10.1016/j.jvb.2010.06.002

[B73] Lyons F. W. Akroyd D. (2014). The impact of human capital and selected job rewards on community college faculty job satisfaction. *Community Coll. J. Res. Pract.* 38 194–207. 10.1080/10668926.2014.851965

[B74] Mahfud T. Triyono M. B. Sudira P. Mulyani Y. (2020). The influence of social capital and entrepreneurial attitude orientation on entrepreneurial intentions: the mediating role of psychological capital. *Eur. Res. Manage. Bus. Econ.* 26 33–39. 10.1016/j.iedeen.2019.12.005

[B75] Man T. W. Lau T. Chan K. (2002). The competitiveness of small and medium enterprises: a conceptualization with focus on entrepreneurial competencies. *J. Bus. Ventur.* 17 123–142. 10.1016/S0883-9026(00)00058-6

[B76] Markman G. D. Baron R. A. (2003). Person–entrepreneurship fit: why some people are more successful as entrepreneurs than others. *Hum. Resour. Manage. Rev.* 13 281–301. 10.1016/S1053-4822(03)00018-4

[B77] Martin B. C. McNally J. J. Kay M. J. (2013). Examining the formation of human capital in entrepreneurship: a meta-analysis of entrepreneurship education outcomes. *J. Bus. Ventur.* 28 211–224. 10.1016/j.jbusvent.2012.03.002

[B78] Marvel M. R. Davis J. L. Sproul C. R. (2016). Human capital and entrepreneurship research: a critical review and future directions. *Entrep. Theory Pract.* 40 599–626. 10.1111/etap.12136

[B79] McArdle S. Waters L. Briscoe J. P. Hall D. T. T. (2007). Employability during unemployment: adaptability, career identity and human and social capital. *J. Vocat. Behav.* 71 247–264. 10.1016/j.jvb.2007.06.003

[B80] Meijers F. Kuijpers M. Gundy C. (2013). The relationship between career competencies, career identity, motivation and quality of choice. *Int. J. Educ. Vocat. Guid.* 13 47–66. 10.1007/s10775-012-9237-4

[B81] Mincer J. (1958). Investment in human capital and personal income distribution. *J. Polit. Econ.* 66 281–302. 10.1086/258055

[B82] Mustafa M. Chen S. (2010). The strength of family networks in transnational immigrant entrepreneurship. *Thunderbird Int. Bus. Rev.* 52 97–106. 10.1002/tie.20317

[B83] Nahapiet J. (2009). “Capitalizing on connections: social capital and strategic management,” in *Social Capital: Reaching Out, Reaching*, eds Bartkus V. O. Davis J. H. (Cheltenham: Edward Elgar), 205–236.

[B84] Newman A. Ucbasaran D. Zhu F. Hirst G. (2014). Psychological capital: a review and synthesis. *J. Organ. Behav.* 35 S120–S138. 10.1002/job.1916

[B85] Ngoma M. Dithan Ntale P. (2016). Psychological capital, career identity and graduate employability in Uganda: the mediating role of social capital. *Int. J. Train. Dev.* 20 124–139. 10.1111/ijtd.12073

[B86] Ostrom E. Ahn T.-K. (2009). “The meaning of social capital and its link to collective action,” in *Handbook of Social Capital: The Troika of Sociology, Political Science and Economics*, eds Svendsen G. T. Svendsen G. L. H. (Northampton, MA: Edward Elgar), 17–35.

[B87] Ozgen E. Minsky B. D. (2006). A perspective into entrepreneurial opportunity recognition in high technology domains: technical competencies as a source of information. *J. Bus. Entrep.* 18 60–73.

[B88] Peng J. Jiang X. Zhang J. Xiao R. Song Y. Feng X. (2013). The impact of psychological capital on job burnout of Chinese nurses: the mediator role of organizational commitment. *PLoS One* 8:e84193. 10.1371/journal.pone.0084193 24416095 PMC3886971

[B89] Petronela T. (2016). The importance of the intangible cultural heritage in the economy. *Procedia Econ. Finance* 39 731–736. 10.1016/S2212-5671(16)30271-4

[B90] Petter S. Straub D. Rai A. (2007). Specifying formative constructs in information systems research. *MIS Q.* 31 623–656. 10.2307/25148814

[B91] Porter L. W. Lawler E. E. (1968). What job attitudes tell about motivation. *Harv. Bus. Rev.* 46 118–126.

[B92] Rego A. Cunha M. P. E. Pinho C. (2009). Exploring a five-factor model of organizational justice. *Manage. Res.* 7 103–125. 10.2753/JMR1536-5433070202

[B93] Requena F. (2003). Social capital, satisfaction and quality of life in the workplace. *Soc. Ind. Res.* 61 331–360. 10.1023/A:1021923520951

[B94] Rözer J. J. Brashears M. E. (2018). Partner selection and social capital in the status attainment process. *Soc. Sci. Res.* 73 63–79. 10.1016/j.ssresearch.2018.03.004 29793692

[B95] Russ F. A. McNeilly K. M. (1995). Links among satisfaction, commitment, and turnover intentions: the moderating effect of experience, gender, and performance. *J. Bus. Res.* 34 57–65. 10.1016/0148-2963(94)00042-D

[B96] Schultz T. W. (1961). Investment in human capital. *Am. Econ. Rev.* 51 1–17.

[B97] Seibert S. E. Kraimer M. L. Liden R. C. (2001). A social capital theory of career success. *Acad. Manage. J.* 44 219–237. 10.5465/3069452 3069452

[B98] Shaffer G. Zhang Z. J. (2002). Competitive one-to-one promotions. *Manage. Sci.* 48 1143–1160. 10.1287/mnsc.48.9.1143.172 19642375

[B99] Skorikov V. Vondracek F. W. (2007). Positive career orientation as an inhibitor of adolescent problem behaviour. *J. Adolesc.* 30 131–146. 10.1016/j.adolescence.2006.02.004 16626800

[B100] Slotte-Kock S. Coviello N. (2010). Entrepreneurship research on network processes: a review and ways forward. *Entrep. Theory Pract.* 34 31–57. 10.1111/j.1540-6520.2009.00311.x

[B101] Sun T. Zhao X. W. Yang L. B. Fan L. H. (2012). The impact of psychological capital on job embeddedness and job performance among nurses: a structural equation approach. *J. Adv. Nurs.* 68 69–79. 10.1111/j.1365-2648.2011.05715.x 21645045

[B102] Taber K. S. (2018). The use of Cronbach’s alpha when developing and reporting research instruments in science education. *Res. Sci. Educ.* 48 1273–1296. 10.1007/s11165-016-9602-2

[B103] Tang J.-J. (2020). Psychological capital and entrepreneurship sustainability. *Front. Psychol.* 11:866. 10.3389/fpsyg.2020.00866 32528347 PMC7248200

[B104] Tenenhaus M. Vinzi V. E. Chatelin Y.-M. Lauro C. (2005). PLS path modeling. *Comput. Stat. Data Anal.* 48 159–205. 10.1016/j.csda.2004.03.005

[B105] Unger J. M. Rauch A. Frese M. Rosenbusch N. (2011). Human capital and entrepreneurial success: a meta-analytical review. *J. Bus. Ventur.* 26 341–358. 10.1016/j.jbusvent.2009.09.004

[B106] Van Dick R. Christ O. Stellmacher J. Wagner U. Ahlswede O. Grubba C. (2004). Should I stay or should I go? Explaining turnover intentions with organizational identification and job satisfaction. *Br. J. Manage.* 15 351–360. 10.1111/j.1467-8551.2004.00424.x

[B107] Vondracek F. W. Skorikov V. B. (1997). Leisure, school, and work activity preferences and their role in vocational identity development. *Career Dev. Q.* 45 322–340. 10.1002/j.2161-0045.1997.tb00537.x

[B108] Wang C. L. Chugh H. (2014). Entrepreneurial learning: past research and future challenges. *Int. J. Manage. Rev.* 16 24–61. 10.1111/ijmr.12007

[B109] Wang I. Shieh C.-J. Wang F.-J. (2008). Effect of human capital investment on organizational performance. *Soc. Behav. Pers.* 36 1011–1022. 10.2224/sbp.2008.36.8.1011

[B110] Wei Y. Liu H. Park K.-S. (2021). Examining the structural relationships among heritage proximity, perceived impacts, attitude and residents’ support in intangible cultural heritage tourism. *Sustainability* 13:8358. 10.3390/su13158358

[B111] West-Burnham J. Farrar M. Otero G. (2007). *Schools and Communities: Working Together to Transform Children’s Lives.* London: A&C Black.

[B112] Wetzels M. Odekerken-Schröder G. Van Oppen C. (2009). Using PLS path modeling for assessing hierarchical construct models: guidelines and empirical illustration. *MIS Q.* 33 177–195. 10.2307/20650284

[B113] Yahya A. Z. Othman C. K. F. A. S. Rahman I. A. Moen J. (2011). Management skills and entrepreneurial success of small and medium enterprises (SMEs) in the services sector. *Afr. J. Bus. Manage.* 5 10410–10418. 10.5897/AJBM11.636

[B114] Zahra S. A. Sapienza H. J. Davidsson P. (2006). Entrepreneurship and dynamic capabilities: a review, model and research agenda. *J. Manage. Stud.* 43 917–955. 10.1111/j.1467-6486.2006.00616.x

[B115] Zhengde X. Yanyan Z. Zhu Y. (2017). The influence of proactive personality on employee’s followership: the mediating role of psychological capital and the moderating role of transformational leadership. *J. Jishou Univ.* 38:43.

